# Supplementary material for: The critical role of natural forest as refugium for generalist species in oil palm-dominated landscapes
Source: PLoS One. 2021 Oct 6;16(10):e0257814. doi: 10.1371/journal.pone.0257814 (PMC8494349; doi:10.1371/journal.pone.0257814)
Supplement: S1 File — (PDF) [file pone.0257814.s001.pdf]

The critical role of natural forest as refugium for generalist species in oil palm-dominated landscapes.

Supplemental material

Table S1. Ranking of POPAN models for Population size estimations (*N*) in program Mark, according with the corrected Akaike information criterion (AICc) value. Φ = survival; *p* = recapture probability; *pent* = entry probability. (·) = no variations; (g) = variations between groups; (t) = variations over time.

Model	AICc	Δ AICc	Weight
{Φ (.) *p* (g) *pent* (t) *N* (g) }	823.9098	0	0.39075
{Φ (t) *p* (g) *pent* (t) *N* (g) }	825.8285	1.9187	0.14971
{Φ (.) *p* (g) *pent* (g) *N* (g) }	825.9173	2.0075	0.14321
{Φ (g) *p* (g) *pent* (t) *N* (g) }	826.3258	2.416	0.11675
{Φ (t) *p* (g) *pent* (g) *N* (g) }	827.945	4.0352	0.05196
{Φ (.) *p* (g) *pent* (g*t) *N* (g) }	828.0084	4.0986	0.05034
{Φ (t) *p*(g*t) *pent* (t) *N* (g) }	828.4279	4.5181	0.04081
{Φ (t) *p* (g) *pent* (g*t) *N* (g) }	829.9438	6.034	0.01913
{Φ (g) *p* (g*t) *pent* (t) *N* (g) }	830.6168	6.707	0.01366
{Φ (.) *p* (g*t) *pent* (g) *N* (g) }	832.7639	8.8541	0.00467
{Φ (g) *p* (t) *pent* (t) *N* (g) }	833.0379	9.1281	0.00407
{Φ (t) *p* (g*t) *pent* (g*t) *N* (g) }	833.2671	9.3573	0.00363
{Φ (g) *p* (g) *pent* (g*t) *N* (g) }	833.3663	9.4565	0.00345
{Φ (g*t) *p* (t) *pent* (t) *N* (g) }	833.954	10.0442	0.00258
{Φ (g) *p* (g) *pent* (g) *N* (g) }	835.3466	11.4368	0.00128
{Φ (.) *p* (g*t) *pent* (t) *N* (g) }	835.9567	12.0469	0.00095
{Φ (g*t) *p* (g*t) *pent* (g*t) *N* (g) }	837.0645	13.1547	0.00054
{Φ (g) *p* (g*t) *pent* (g*t) *N* (g) }	837.1141	13.2043	0.00053
{Φ (.) *p* (t) *pent* (t) *N* (g) }	838.318	14.4082	0.00029
{Φ (g*t) *p* (g*t) *pent* (t) *N* (g) }	838.403	14.4932	0.00028
{Φ (.) *p* (g*t) *pent* (g*t) *N* (.) }	839.6435	15.7337	0.00015

**{Φ (t) *p* (g*t) *pent* (g*t) *N* (.) }**	839.9932	16.0834	0.00013
**{Φ (g) *p* (g*t) *pent* (g*t) *N* (.) }**	840.0581	16.1483	0.00012
**{Φ (t) *p* (t) *pent* (t) *N* (g) }**	840.5078	16.598	0.0001
**{Φ (g) *p* (.) *pent* (g) *N* (g) }**	840.6465	16.7367	0.00009
**{Φ (g*t) *p* (g) *pent* (g*t) *N* (g) }**	840.7822	16.8724	0.00008
**{Φ (g) *p* (t) *pent* (g*t) *N* (g) }**	840.8953	16.9855	0.00008
**{Φ (g*t) *p* (g) *pent* (g) *N* (g) }**	840.9479	17.0381	0.00008
**{Φ (g) *p* (.) *pent* (g*t) *N* (g) }**	840.9687	17.0589	0.00008
**{Φ (g) *p* (t) *pent* (g) *N* (g) }**	840.9938	17.084	0.00008
**{Φ (g) *p* (g*t) *pent* (g) *N* (g) }**	841.2733	17.3635	0.00007
**{Φ (g*t) *p* (t) *pent* (g*t) *N* (g) }**	842.1494	18.2396	0.00004
**{Φ (g) *p* (t) *pent* (t) *N* (.) }**	842.2749	18.3651	0.00004
**{Φ (t) *p* (g*t) *pent* (t) *N* (.) }**	842.2879	18.3781	0.00004
**{Φ (.) *p* (g*t) *pent* (g*t) *N* (g) }**	842.8266	18.9168	0.00003
**{Φ (.) *p* (g*t) *pent* (t) *N* (.) }**	843.7158	19.806	0.00002
**{Φ (g) *p* (g) *pent* (t) *N* (.) }**	843.8202	19.9104	0.00002
**{Φ (t) *p* (t) *pent* (g*t) *N* (g) }**	843.9997	20.0899	0.00002
**{Φ (g*t) *p* (g*t) *pent* (g) *N* (g) }**	844.2262	20.3164	0.00002
**{Φ (g*t) *p* (g) *pent* (t) *N* (g) }**	844.2875	20.3777	0.00001
**{Φ (t) *p* (t) *pent* (g) *N* (g) }**	844.4546	20.5448	0.00001
**{Φ (g*t) *p* (g*t) *pent* (g*t) *N* (.) }**	844.7163	20.8065	0.00001
**{Φ (.) *p* (t) *pent* (g) *N*(g) }**	844.7359	20.8261	0.00001
**{Φ (.) *p* (.) *pent* (g) *N* (g) }**	844.8757	20.9659	0.00001
**{Φ (g*t) *p* (.) *pent* (g) *N* (g) }**	845.1726	21.2628	0.00001
**{Φ (g) *p* (g*t) *pent* (t) *N* (.) }**	845.4359	21.5261	0.00001
**{Φ (.) *p* (.) *pent* (g*t) *N* (g) }**	845.6579	21.7481	0.00001
**{Φ (.) *p* (t) *pent* (g*t) *N* (g) }**	845.663	21.7532	0.00001
**{Φ (g*t) *p* (g*t) *pent* (t) *N* (.) }**	846.6081	22.6983	0
**{Φ (t) *p* (.) *pent* (g) *N* (g) }**	846.8975	22.9877	0
**{Φ (g*t) *p* (t) *pent* (t) *N* (.) }**	847.2983	23.3885	0
**{Φ (.) *p* (t) *pent* (t) *N* (.) }**	847.5136	23.6038	0
**{Φ (t) *p* (.) *pent* (g*t) *N* (g) }**	847.6209	23.7111	0
**{Φ (g*t) *p* (g) *pent* (t) *N* (.) }**	847.8142	23.9044	0

**{Φ (g) *p* (t) *pent* (g*t) *N* (.) }**	848.0706	24.1608	0
**{Φ (t) *p* (g*t) *pent* (g) *N* (g) }**	848.3417	24.4319	0
**{Φ (.) *p* (g*t) *pent* (g) *N* (.) }**	848.4958	24.586	0
**{Φ (t) *p* (t) *pent* (t) *N* (.) }**	849.1897	25.2799	0
**{Φ (g) *p* (g*t) *pent* (g) *N* (.) }**	849.4912	25.5814	0
**{Φ (g*t) *p* (g*t) *pent* (g) *N* (.) }**	851.8501	27.9403	0
**{Φ (.) *p* (t) *pent* (g*t) *N* (.) }**	852.8064	28.8966	0
**{Φ (g) *p* (.) *pent* (t) *N* (g) }**	852.9074	28.9976	0
**{Φ (g*t) *p* (t) *pent* (g*t) *N* (.) }**	853.0155	29.1057	0
**{Φ (g) *p* (g) *pent* (g*t) *N* (.) }**	853.5505	29.6407	0
**{Φ (g) *p* (g) *pent* (g) *N* (.) }**	854.295	30.3852	0
**{Φ (t) *p* (t) *pent* (g*t) *N* (.) }**	854.3141	30.4043	0
**{Φ (g*t) *p* (g) *pent* (g*t) *N* (.) }**	857.3459	33.4361	0
**{Φ (g) *p* (t) *pent* (g) *N* (.) }**	859.7631	35.8533	0
**{Φ (.) *p* (g) *pent* (g*t) *N* (.) }**	860.2042	36.2944	0
**{Φ (g*t) *p* (.) *pent* (t) *N* (g) }**	861.0104	37.1006	0
**{Φ (.) *p* (g) *pent* (g) *N* (.) }**	861.5992	37.6894	0
**{Φ (t) *p* (g) *pent* (g*t) *N* (.) }**	862.1718	38.262	0
**{Φ (g) *p* (.) *pent* (t) *N* (.) }**	863.6276	39.7178	0
**{Φ (t) *p* (g) *pent* (g) *N* (.) }**	863.6533	39.7435	0
**{Φ (t) *p* (.) *pent* (t) *N* (g) }**	864.4453	40.5355	0
**{Φ (g*t) *p* (t) *pent* (g) *N* (.) }**	864.9747	41.0649	0
**{Φ (.) *p* (t) *pent* (g) *N* (.) }**	865.1147	41.2049	0
**{Φ (t) *p* (t) *pent* (g) *N* (.) }**	866.9715	43.0617	0
**{Φ (t) *p* (g*t) *pent* (g) *N* (.) }**	868.7532	44.8434	0
**{Φ (.) *p* (.) *pent* (t) *N* (.) }**	868.8003	44.8905	0
**{Φ (g*t) *p* (.) *pent* (t) *N* (.) }**	868.8313	44.9215	0
**{Φ (g) *p* (.) *pent* (g*t) *N* (.) }**	870.7836	46.8738	0
**{Φ (g*t) *p* (t) *pent* (g) *N* (g) }**	871.8674	47.9576	0
**{Φ (g) *p* (.) *pent* (g) *N* (.) }**	874.4532	50.5434	0
**{Φ (.) *p* (.) *pent* (g*t) *N* (.) }**	875.8345	51.9247	0
**{Φ (g*t) *p* (.) *pent* (g*t) *N* (.) }**	876.1389	52.2291	0
**{Φ (t) *p* (.) *pent* (g*t) *N* (.) }**	877.882	53.9722	0

**{Φ (g*t) *p* (.) *pent* (g) *N* (.) }**	879.7201	55.8103	0
**{Φ (.) *p* (g) *pent* (t) *N* (.) }**	879.9857	56.0759	0
**{Φ (t) *p* (.) *pent* (t) *N* (.) }**	880.7939	56.8841	0
**{Φ (t) *p* (.) *pent* (g) *N* (.) }**	881.5418	57.632	0
**{Φ (t) *p* (g) *pent* (t) *N* (.) }**	882.0074	58.0976	0
**{Φ (g*t) *p* (g) *pent* (g) *N* (.) }**	891.519	67.6092	0

Table S2. Ranking of Pradel's models for population growth (λ) and survival (Φ) estimations in program Mark, according with the corrected Akaike information criterion (AICc) value. ΔAICc = Difference with the lower AICc; *p* = recapture probability. (·) = no variations; (g) = variations between groups; (t) = variations over time.

Model	AICc	Δ AICc	Weights
**{Φ (.) *p* (g) λ (g) }**	2152.6293	0	0.28119
**{Φ (t) *p* (g) λ (g) }**	2153.7416	1.1123	0.16124
**{Φ (t) *p* (g) λ (g) }**	2153.7416	1.1123	0.16124
**{Φ (g) *p* (.) λ (g) }**	2157.543	4.9137	0.0241
**{Φ (g) *p* (.) λ (g) }**	2157.543	4.9137	0.0241
**{Φ (g) *p* (g) λ (g) }**	2157.8021	5.1728	0.02117
**{Φ (g) *p* (g) λ (g) }**	2157.8021	5.1728	0.02117
**{Φ (g) *p* (t) λ (g) }**	2159.8357	7.2064	0.00766
**{Φ (g) *p* (t) λ (g) }**	2159.8357	7.2064	0.00766
**{Φ (.) *p* (.) λ (g) }**	2162.432	9.8027	0.00209
**{Φ (.) *p* (.) λ (g) }**	2162.432	9.8027	0.00209
**{Φ (t) *p* (.) λ (g) }**	2163.6021	10.9728	0.00116
**{Φ (t) *p* (.) λ (g) }**	2163.6021	10.9728	0.00116
**{Φ (.) p(t) λ (g) }**	2164.6501	12.0208	0.00069
**{Φ (t) p(t) λ (g) }**	2164.6501	12.0208	0.00069
**{Φ (.) p(t) λ (g) }**	2164.6501	12.0208	0.00069
**{Φ (t) p(t) λ (g) }**	2164.6501	12.0208	0.00069
