# Supplementary material for: The critical role of natural forest as refugium for generalist species in oil palm-dominated landscapes
Source: PLoS One. 2021 Oct 6;16(10):e0257814. doi: 10.1371/journal.pone.0257814 (PMC8494349; doi:10.1371/journal.pone.0257814)
Supplement: Attachment — Submitted filename: Response to Editor.docx [file pone.0257814.s002.docx]

Dear Editor:

Thank you very much for your inputs and comments on our manuscript.

We have responded to every one of the comments made by the reviewers and addressed the recommendations accordingly.

In regards to your comment on Figure 1, the map was produced by the first author (Guerrero-Sanchez) with geographic information (forest and plantation layers) provided by the Danau Girang Field Centre, we have included a mention to the officer, Lucy P. Liew in the acknowledgements:

“*The map in figure 1 was generated by the authors with geographical information (Forest and plantation layers) provided by Lucy P. Lew, from the Danau Girang Field Centre”* (line 403 – 405)

The trapping sites and “suitable habitat” was generated by Guerrero-Sanchez with information generated by himself.

We hope this help to avoid confusions on copy right.
