# Supplementary material for: The critical role of natural forest as refugium for generalist species in oil palm-dominated landscapes
Source: PLoS One. 2021 Oct 6;16(10):e0257814. doi: 10.1371/journal.pone.0257814 (PMC8494349; doi:10.1371/journal.pone.0257814)
Supplement: Attachment — Submitted filename: Response to Reviewer2.docx [file pone.0257814.s003.docx]

**Responses to Reviewer 2:**

Although you have partially addressed my previous concerns, I feel some still remain unaddressed in your responses. My remaining concerns are as follows:

"**R2-C2**. Lines 48-54: Distance sampling and mark-recapture actually provide different types of information so I think it is less about accuracy and more about the purpose of the survey."
In your response you state that Distance Sampling cannot account for variation in detectability between the two habitats, but this is strictly untrue. This is exactly what Distance Sampling is designed to do and I suggest you modify this statement.

RE: The paragraph has been modified as follows:

“Earlier studies on the species’ populations focused on determining the differences on abundance between natural forest and human-modified habitats using either distance surveys or mark-recapture methods. However, a more detailed information is needed to properly evaluate the status of a population, especially when focusing on species that are target for either commercial or subsistence consumption” (Actual lines 55-59).

"**R2-C3**. Sampling design. Need more information about the sampling design and how you
matched plantation and forest plots to control for confounding variables (for example, why are all the forest plots along the river? This is likely to bias your results)."
In your response you seem to explain how you achieved independence among sampling sites not how you dealt with confounding factors. I understand that accounting for confounding factors may be difficulty, but at the very least it needs to be discussed as a major limitation (if they can’t be dealt with) and provide an explanation that, if there are unaccounted for confounding factors, the ability to generalise your results to other systems will be limited (i.e., making general statements about the differences between oil palm and natural forest will not be possible).

RE: We added a paragraph in the methods section acknowledging the limitations of the sampling method and we recommended to take the outcomes of the study with caution:

“However, although transects in both forest and oil palm areas were meant to be spatially standardized, traps in forest transects along the main river may differ regarding trapping success, with respect to traps in transects at the edge of the plantations. Such effects may reflect the size of water bodies present in the area (e.g. main river in forested areas v. tributary rivers and drains along the edge of plantation areas). While this issue was unavoidable due to the spatial conformation of the Lower Kinabatangan Wildlife Sanctuary and the size and shape of forest fragments remaining, such sampling design limitations should be taken into consideration when interpreting the results, and when replicating the study in different geographical areas.” (Actual lines 143-151).

"**R2-C4**. Statistical analysis. What is the spatial unit for which you estimated population size? Is it the transect? This should be made clear."
In your modified paragraph what does “higher probability of occurrence” really mean? I think you need to be much more specific here. I assume you set a threshold that defined suitable habitat and if this is what you did then that threshold needs to be defined.

RE: We defined the area with “higher probability of occurrence” as the area generated by the MAXENT model with a probability ratio of occurrence above the unit (PRO ≥ 1). It is the output created by the predictive model where 1 represents the probability of occurrence in an “average” location in the training data, while any X value above the unit represent X times higher than the average probability of presence (e.g. a 2 would imply a twice as high probability of presence). The paragraph was modified as follows:

“The number of individuals estimated in the model would correspond to the sampling area, which is the area covered by the trap influence areas in each trapping site. Such numbers were extrapolated to a suitable area, defined as the area where the probability of presence of the species is relatively higher than the probability value in an average location in the training data (Probability Ratio Outcome; PRO > 1), with values larger than 1 representing the increase in likelihood of finding an animal (e.g. 2 corresponds to twice as likely). Maximum Entropy (MAXENT) models for habitat selection with telemetry data (unpublished) were used to estimate the areas of higher probability of occurrence (PRO≥ 1). The same suitable area was also used to estimate the actual population density (ind./km^2^).” (Actual lines 177-186).

"**R2-C8**. Lines 200-204. You cannot compare the AIC of models fitted to different data sets (which seems to be what you are doing here unless I am mistaken)."
In your response you never answer my question about whether you compare AIC values for models fitted to different data sets. It still sounds like you did, which would be incorrect if so.

Re: We have modified the respective paragraph as follows:

“GLS models were tested to assess the effects of morphometric variables (body weight and length), and suitable area, on the population size in each habitat type. We observed that the size of suitable habitat in forested areas has a positive effect on the population size (GLS = 28.44 ± 6.62; t = 4.30; p < 0.001), but not in oil palm plantations. Instead, in these anthropogenic habitats, body length has a significant negative effect on the number of individuals in the population (GLS = -1.05 ± 0.49; t = -2.13; p = 0.034)”. (Actual lines 258-263; Table 3 has been modified too).
